# Les syndromes coronaires aigus à Dakar: aspects cliniques thérapeutiques et évolutifs

**DOI:** 10.11604/pamj.2014.19.126.3155

**Published:** 2014-10-03

**Authors:** Mouhamed Cherif Mboup, Maboury Diao, Khadidiatou Dia, Pape Diadie Fall

**Affiliations:** 1 Departement de Cardiologie, Hôpital Principal de Dakar, Sénégal

**Keywords:** Syndromes coronaires aigus, facteurs de risque cardio-vasculaires, Dakar, acute coronary syndromes, cardiovascular risk factors, Dakar

## Abstract

**Introduction:**

Ce travail a pour objectifs d’étudier les aspects épidémiologiques et cliniques, les différentes modalités de prise en charge, et l’évolution des syndromes coronaires aigus à Dakar.

**Méthodes:**

Il s'agit d'une étude prospective concernant une cohorte de patients hospitalisés au niveau des services de cardiologie de l'hôpital Principal de Dakar et de l'hôpital Aristide Le Dantec pour un syndrome coronaire aigu entre le 01 Septembre 2005 et le 31 Août 2006.

**Résultats:**

Durant la période d’étude, 59 patients avaient présenté à l'admission un syndrome coronaire aigu, soit une prévalence hospitalière de 4,05%. L’âge moyen était de 57,1 ± 3,5 ans. L'indice moyen des facteurs de risque était de 3,56 ± 1,7. Quatre vingt onze pour cent (91%) des patients avaient au moins deux facteurs de risque cardio-vasculaires. Les délais moyens d'arrivée et de prise en charge étaient respectivement de 53,2 ± 21,3 heures et 3,4 ± 1 heures. L'analyse des tracés électrocardiographiques associée au dosage des troponines, avait permis de retenir les diagnostics d'infarctus du myocarde avec sus-décalage persistant du segment ST chez 89,8% patients, d'infarctus du myocarde sans sus-décalage du segment ST chez 5,1% patients, et d'angor instable dans 5,1% des cas. La mortalité hospitalière était de 15,25% et la mortalité à un mois de 18,64%.

**Conclusion:**

Au Sénégal, les syndromes coronaires aigus sont caractérisés par un âge de survenue relativement jeune chez des patients polyfactoriels et une lourde mortalité. C'est dire tout l'intérêt d'une prévention primaire efficace par la lutte contre les facteurs de risque cardio-vasculaires.

## Introduction

Le syndrome coronaire aigu regroupe des présentations cliniques dont la prise en charge en phase aiguë diffère selon qu'il existe ou non un sus-décalage du segment ST à l’électrocardiogramme [1, 2]. Première cause de mortalité dans les pays industrialisés [1], en Afrique, sa prévalence connaît une augmentation en rapport avec l'occidentalisation des habitudes de vie, l'amélioration des conditions socio-économiques et l'augmentation de l'espérance de vie [3]. L’émergence de ces affections dans nos pays contraste avec une insuffisance du plateau technique de cardiologie interventionnelle indispensable à une prise en charge efficiente des patients. Dans le cadre de la prise en charge des syndromes coronaires aigus au Sénégal, nous avons réalisé une étude prospective multicentrique sur un an avec comme objectifs: étudier les aspects épidémiologiques et cliniques des syndromes coronaires aigus; apprécier les différentes modalités de prise en charge pré-hospitalière et hospitalière; évaluer l'impact de cette prise en charge sur l’évolution.

## Méthodes

Il s'agit d'une étude transversale, prospective qui a colligé des patients hospitalisés au niveau des services de cardiologie de l'hôpital Principal de Dakar et de l'hôpital Aristide Le Dantec pour un syndrome coronaire aigu (SCA) entre le 1er septembre 2005 et le 31 août 2006. Nous avions inclus tous les patients présentant: une douleur angineuse au repos et ou; des modifications électrocardiographiques à type de sus-décalage ou de sous-décalage significatifs du segment ST, ou des ondes T négatives symétriques. On avait exclu les patients présentant un angor stable, une douleur thoracique et/ou des modifications électrocardiographiques non spécifiques, un infarctus du myocarde semi récent ou séquellaire. Les paramètres étudiés étaient: les éléments épidémiologiques: la prévalence, l’âge, le sexe, les antécédents, les moyens et modes de transport, les délais d'admission et de prise en charge; les aspects cliniques et paracliniques (biologie, électrocardiogramme, échocardiographie Doppler); les modalités de prise en charge, l’évolution intra-hospitalière et à un mois. Nous avions réalisé une analyse statistique avec le logiciel SPSS 11.0. Le test de Khi 2 était utilisé et était considéré comme significatif pour une valeur de p < 0,05.

## Résultats

Sur 1457 patients hospitalisés, 59 avaient présenté à l'admission un syndrome coronaire aigu (SCA), soit une prévalence hospitalière de 4,05%. L’âge moyen était de 57,1 ± 3,5 ans (extrêmes de 31 et 85 ans). Les tranches d’âge les plus représentatives étaient celles comprises entre 51-60 ans (23,7%) et 61-70 ans (23,7%). Six patients (10,1%) étaient âgés de moins de 40 ans. On notait une prédominance masculine avec un sex-ratio de 3,9. Dans les antécédents, on retrouvait un angor stable et un infarctus du myocarde respectivement dans 33,9% et 1,7%. Quatre vingt onze pour cent (91%) des patients avaient au moins deux facteurs de risque cardio-vasculaire. L'indice moyen des facteurs de risque était de 3,56 ± 1,7 (extrêmes de 0 et 8). Les moyens de transport étaient: la voiture personnelle (49,2%), les transports en commun (39%), l'ambulance non médicalisée (11,9%). Trente quatre patients (57,6%) étaient admis directement aux urgences des structures hospitalières servant de cadre à notre travail. Les autres patients (42,4%) avaient fait l'objet d'un passage préalable dans des structures sanitaires périphériques. Les patients consultaient en moyenne 1,3 ± 1 structure sanitaire périphérique avant leur admission à l'hôpital (extrêmes de 1 et 3). Le délai moyen d'arrivée était de 53,2 ± 21,3 heures (extrêmes d'1 et 336 heures). Vingt trois patients (38,9%) présentant un SCA avec sus-décalage du segment ST étaient reçus dans les 12 premières heures. Pour les patients admis directement dans les hôpitaux, le délai moyen d'arrivée était de 30,7 ± 17,6 heures (extrêmes de 1 et 240 heures) et quant à ceux transférés à partir d'une structure sanitaire périphérique, ce délai était de 86,6 ± 43,3 heures (extrêmes de 2 et 336 heures) avec une différence statistiquement significative (p = 0,018). La prise en charge cardiologique était effective en moyenne au bout de 3,4 ± 1 heures (extrêmes de 30 minutes et 48 heures) après leur arrivée à l'hôpital. La symptomatologie était dominée par un angor (79,7%), Les autres types de douleur étaient une épigastralgie et une douleur basithoracique respectivement (3,38%), une piqûre rétrosternale (8,46%) et une douleur rétrosternale à type de coup de poignard (1,7%). Deux patients (3,38%) étaient asymptomatiques. Cette symptomatologie douloureuse était accompagnée de nausées et vomissements dans 44,1% des cas.

L’électrocardiogramme inscrivait un infarctus antérieur (61%), inférieur (33,9%), circonférentiel (3,4%) et une extension au ventricule droit (13,6%). Le Tableau 1 représente la topographie des anomalies ECG au cours des SCA. Les résultats de la biologie sont résumés dans leTableau 2. La troponine, dosée chez 48 patients (81,3%) n’était inférieure à 0,5ng/ml que dans 3 cas. La glycémie dosée dans 95% des cas, était en moyenne de 1,62 ± 0,29g/l (extrêmes de 0,5 et 4,7g/l). Vingt deux patients (37,2%) avaient une glycémie supérieure à 1,24g/l. Au plan lipidique, vingt patients (33,9%) avaient un cholestérol total au-delà de 2g/l, quatre patients (6,8%) un LDL cholestérol supérieur à 1,6 g/l et 16 malades (27,1%) un HDL cholestérol inférieur à 0,4 g/l. Dans treize cas (22%), les triglycérides étaient supérieurs à 1,5 g/l. Les anomalies de la troponine, associées à la symptomatologie, avaient permis de retenir les diagnostics de: SCA avec sus-décalage persistant du segment ST (89,8%); SCA sans sus-décalage du segment ST (10,2%) répartis respectivement en infarctus du myocarde sans sus décalage de ST (5,1%) et angor instable (5,1%).

**Tableau 1:** Diagnostic topographique des syndromes coronariens à l'ECG

Syndromes Coronaires aigus		Infarctus du myocarde avec sus-décalage du segment ST	Infarctus du myocarde sans sus-décalage du segment ST	Angor instable
Territoires				
Antérieur	Etendu	19 (32,2%)	-	-
	Antéro-Septal	5 (8,5%)	-	-
	Antéro-septo-apical	6 (10,2%)	-	-
	Apico-latéral	6 (10,2%)	3 (5,1%)	-
Postéro-diaphragmatique		20 (33,9%)	-	3 (5,1%)
Circonférentiel		2 (3,4%)	-	-
Extension au ventricule droit d'un infarctus inférieur		8 (13,6%)	-	-

**Tableau 2:** Paramètres biologiques

Paramètres biologiques		Valeurs moyennes	Extrêmes
Troponines (ng/ml)		18,5 ± 8,2	0,03 – 146,05
Créatines Phospho-kinases (CPK) (UI/l)	Totales	11489,7 ± 672,3	33 – 9999
	Mb	162,2 ± 56,8	
	CPKTotales/Mb	14,7 ± 3,3	3 – 38,3
Glycémie (g/l)		1,6 ± 0,29	0,5 – 4,7
Cholestérol (g/l)	Total	1,76 ± 0,1	0,5 – 3,4
	LDL	0,96 ± 0,13	0,1 – 3
	HDL	0,49 ± 0,05	0,1 -1
Triglycérides (g/l)		1,19 ± 0,15	0,1 – 2,8

Les anomalies échocardiographiques étaient: des troubles de la cinétique segmentaire (92,7%), une altération de la fonction systolique VG avec FE inférieure à 50% (32%). Un thrombus intra-ventriculaire gauche était retrouvé dans 3 cas (5,1%). Les anomalies échocardiographiques sont résumées dans le Tableau 3. Au plan thérapeutique, 6 patients (10,2%) avaient bénéficié d'une thrombolyse réalisée 5,2 ± 1,3 heures (extrêmes de 1 et 11 heures) après le début de la douleur. Les autres médicaments utilisés sont récapitulés dans le Tableau 4. L’évolution en milieu hospitalier était favorable dans 52,5% des cas. Des complications étaient survenues dans 47,5% des cas à type d'insuffisance cardiaque (16,9%). Les complications sont récapitulées par le Tableau 5. L’épreuve d'effort, réalisée au delà du dixième jour sous traitement, était positive chez 12,5% des patients. La mortalité hospitalière était de 15,2% et celle à un mois de 18,6%. En analyse bivariée, une fraction d’éjection ventriculaire gauche inférieure à 50% (p = 0,03) et l'insuffisance cardiaque (Killip supérieure à I) (p = 0,001) constituaient des facteurs prédictifs de mortalité à un mois. Les patients avec un bloc auriculo-ventriculaire complet avaient bénéficié d'implantation d'une sonde de stimulation temporaire et d'un pacemaker définitif respectivement 2 cas.

**Tableau 3:** Anomalies écho cardiographiques

Anomalies écho cardiographiques		Nombre de patients	Pourcentage
Cinétique	Hypo kinésie	32	54%
	Akinésie	19	32%
	Dyskinésie	4	6,7%
Cardiomyopathies	Ischémiques dilatées	11	18,6%
	Hypertrophiques	9	15,2%
Fraction d’éjection du ventricule gauche inférieure à 50%		19	32%
Flux de régurgitation	Insuffisance mitrale	18	30,5%
	Insuffisance tricuspide	4	6,7%
	Insuffisance aortique	1	1,7%
Trouble de la relaxation du ventricule gauche		5	8,4%
Thrombus intra-ventriculaire gauche		3	5,1%

**Tableau 4:** Les médicaments utilisés

Médicaments	Nombre de Patients	Pourcentage
Héparines de bas poids moléculaire	57	96,6%
Aspirine	53	89,9%
Inhibiteurs Enzyme Conversion	47	79,7%
Bêtabloquants	41	69,5%
Statines	32	54,2%
Anxiolytiques	18	30,5%
Cimetidine	14	23,7%
Diurétiques	10	16,9%
Antalgiques	10	16,9%
Insuline	8	13,5%
Clopidogrel	7	11,8%
Streptokinase	6	10,2%
Dérivés Nitrés	5	8,47%
Acenocoumarol	3	5,1%
Inhibiteur Calcique	1	1,7%
Antidiabétique Oral	1	1,7%
Fibrate	1	1,7%
Cordarone	1	1,7%
Dobutamine	1	1,7%

**Tableau 5:** Les complications

Complications		Nombre de Patients	Pourcentage
Insuffisance cardiaque		10	16,9%
Troubles de la conduction	Bloc de Branche droit Complet	4	6,7%
	Bloc auriculo-ventriculaire II degré type I	1	1,7%
	Bloc auriculo-ventriculaire II degré type II	2	3,4%
	Bloc auriculo-ventriculaire Complet	5	8,4%
Récidive douloureuse		3	5,1%
Tachycardie ventriculaire		2	3,4%
Choc cardiogénique		2	3,4%
Accident vasculaire cérébral ischémique		1	1,7%

## Discussion

En Afrique subsaharienne, on constate une progression régulière de la prévalence des affections coronariennes. Celle-ci est passée de 3,17% en 1991 dans l'enquête prospective multicentrique CORONAFRIC [4] à 4,05% à Dakar en 2006. Elles représentent 6,2% des urgences cardio-vasculaires [5]. L'augmentation de la prévalence de ces affections s'accompagne d'une diminution de l’âge moyen de survenue de 57,1 ± 3,5 ans au Sénégal et 55,5 ± 11,6 ans en Tunisie [6]. Dans les séries européennes et nord-américaines, l’âge moyen de survenue des syndromes coronaires aigus est beaucoup plus élevé. Il est de 67 ± 14 ans, 62,6 ans et 65 ± 13 ans respectivement en France, au Canada et en Grèce [7–9]. Cette disparité pourrait s'expliquer d'une part par l'espérance de vie beaucoup plus élevée dans ces pays, mais surtout par l'absence de programmes efficaces de lutte contre les facteurs de risque cardio-vasculaires en Afrique. Ainsi, la présence d'au moins un facteur de risque est la règle en matière de cardiopathie ischémique [10]. Koate en 1981 signalait la forte prédominance des cardiopathies ischémiques bifactorielles 49,3%, par rapport aux trifactorielles 23% et quadrifactorielles 4,7% [10]. Cependant, il prévoyait que dans les années à venir, les formes trifactorielles et même quadrifactorielles ne deviennent prépondérantes [10]. Notre étude réalisée vingt cinq années plus tard, confirme cette prédiction en mettant en évidence une égalité de la prévalence des cardiopathies ischémiques à deux, trois, quatre et cinq facteurs de risque.

La voiture personnelle (49,2%) constitue le moyen de transport le plus fréquemment utilisé par nos patients pour se rendre au niveau des services d'accueil des urgences des hôpitaux. Les structures préhospitalières sont exceptionnellement sollicitées. Cette stratégie est différente de celle retrouvée dans les pays européens, où les intervenants préhospitaliers représentés essentiellement par le service de SMUR sont de plus en plus sollicités ces dernières années [11]. Cette différence est essentiellement liée à l'absence de sensibilisation des populations africaines sur les affections cardio-vasculaires en général et sur la nécessité de recourir aux structures préhospitalières devant toute douleur thoracique en particulier. Cependant le coût élevé de la prestation de ces structures et leur disponibilité pourraient constituer un frein à cette sensibilisation. Les moyens de transport utilisés par les patients concourent à allonger les délais d'admission au niveau des services d'accueil des urgences. Il est de 53,2 heures, 14 heures et 4 heures respectivement au Sénégal, en Tunisie [12] et en Grèce [9]. Mahdhaoui et al avait montré en analyse multivariée que le diabète et surtout une symptomatologie atypique constituaient des facteurs indépendants associés à un délai d'admission supérieur à 12 heures [12]. Par rapport à l'heure d'arrivée, nos patients bénéficiaient d'une prise en charge cardiologique au bout de 3,4 ± 1 heures. En Tunisie, Mahdhaoui et al retrouvaient des délais de prise en charge sensiblement comparables (2,3 ± 8,25 heures) [12]. Cet allongement des délais de prise en charge imputable aux médecins constitue un obstacle à la mise en œuvre d'une stratégie de reperfusion myocardique par thrombolyse. Ces constatations nous amènent à réfléchir sur toute la chaîne de prise en charge des syndromes coronaires aigus, mais surtout sur la nécessité d'une formation médicale continue des praticiens exerçant au niveau des services d'accueil des urgences des hôpitaux africains.

Notre étude met en évidence une prépondérance de l'infarctus du myocarde avec sus-décalage persistant du segment ST (89,8%) sur les autres formes cliniques de syndromes coronaires aigus. Cette répartition diffère de celle décrite dans la littérature. Classiquement, l'angor instable représente plus du tiers (37%) des syndromes coronaires aigus hospitalisés, l'infarctus du myocarde avec sus-décalage du segment ST 35%, le reste des patients présentant un infarctus du myocarde sans sus-décalage du segment ST [1, 2].

La dysfonction systolique du ventricule gauche individualisée chez 32,2% de nos patients constitue un facteur de mauvais pronostique comme l'avaient soulignée Cambou et al dans l’étude USIK [2]. Dans ce travail, 54% des patients avaient une fraction d’éjection ventriculaire gauche inférieure à 50%. Celle-ci constituait en analyse multivariée, un facteur prédictif de mortalité en 1 an [7]. Cette dysfonction systolique est due à la diminution de la masse contractile en rapport avec l'akinésie du territoire nécrosé et des zones ischémiques, notamment périnécrotiques. Ainsi, l'insuffisance ventriculaire gauche précoce est souvent réversible, car le territoire akinétique peut être sidéré ou simplement ischémié, et récupéré en quelques semaines, en cas de revascularisation précoce et complète [13]. C'est dire tout l'intérêt d'une amélioration du plateau technique des hôpitaux de Dakar pour une prise en charge plus efficiente des patients.

Nous avons enregistré une lourde mortalité de 18,64% à un mois. Celle-ci est de 13,2% en France dans l’étude USIK [7]. Il existe un gradient de mortalité sud-nord inhérent: à notre plateau technique, caractérisé par l'absence de salle de coronarographie, de chirurgie de revascularisation, et d'inhibiteurs des récepteurs à la glycoprotéine IIb/IIIa; aux longs délais de prise en charge limitant l'instauration d'un traitement thrombolytique, qui demeure la seule méthode de revascularisation disponible dans nos hôpitaux.

## Conclusion

Les syndromes coronaires aigus constituent des affections de plus en plus fréquentes en Afrique subsaharienne. Au Sénégal, ils sont caractérisés par un âge de survenue relativement jeune chez des patients polyfactoriels. La prise en charge de ces affections pose plusieurs problèmes inhérents aux longs délais de consultation et de prise en charge des malades, aux structures préhospitalières peu connues des populations, et à l'absence de salle de coronarographie. L'amélioration de la prise en charge de ces affections dans nos pays en voie de développement passe par une prévention primaire efficiente par des campagnes d'information, de sensibilisation, et de lutte contre les facteurs de risque cardio-vasculaires.
